# Chemical and Molecular Characterization of Wound-Induced Suberization in Poplar (*Populus alba* × *P. tremula*) Stem Bark

**DOI:** 10.3390/plants11091143

**Published:** 2022-04-22

**Authors:** Meghan K. Rains, Christine Caron, Sharon Regan, Isabel Molina

**Affiliations:** 1 Department of Biology, Algoma University, Sault Ste. Marie, ON P6A 2G4, Canada; 14mkr1@queensu.ca (M.K.R.); christinecaron76@gmail.com (C.C.); 2 Department of Biology, Queen’s University, Kingston, ON K7L 3N6, Canada; regans@queensu.ca

**Keywords:** *Populus* spp., bark, wound periderm, lipid polyesters, suberin, lignin, aliphatic and phenolic monomers, GC–MS, GC-FID, TEM

## Abstract

Upon mechanical damage, plants produce wound responses to protect internal tissues from infections and desiccation. Suberin, a heteropolymer found on the inner face of primary cell walls, is deposited in specific tissues under normal development, enhanced under abiotic stress conditions and synthesized by any tissue upon mechanical damage. Wound-healing suberization of tree bark has been investigated at the anatomical level but very little is known about the molecular mechanisms underlying this important stress response. Here, we investigated a time course of wound-induced suberization in poplar bark. Microscopic changes showed that polyphenolics accumulate 3 days post wounding, with aliphatic suberin deposition observed 5 days post wounding. A wound periderm was formed 9 days post wounding. Chemical analyses of the suberin polyester accumulated during the wound-healing response indicated that suberin monomers increased from 0.25 to 7.98 mg/g DW for days 0 to 28, respectively. Monomer proportions varied across the wound-healing process, with an overall ratio of 2:1 (monomers:glycerol) found across the first 14 days post wounding, with this ratio increasing to 7:2 by day 28. The expression of selected candidate genes of poplar suberin metabolism was investigated using qRT-PCR. Genes queried belonging to lipid polyester and phenylpropanoid metabolism appeared to have redundant functions in native and wound-induced suberization. Our data show that, anatomically, the wounding response in poplar bark is similar to that described in periderms of other species. It also provides novel insight into this process at the chemical and molecular levels, which have not been previously studied in trees.

## 1. Introduction

Plants are subjected to a number of biotic and abiotic stresses throughout their lifecycle, which may damage external tissues and expose the plant to disease or desiccation. Biopolymers such as cutin and suberin are cell wall modifications present in primary and secondary boundary tissues, respectively, forming protective physical barriers that represent constitutive defense mechanisms against injuries. When any of these barriers are disrupted, the ability of plants to employ strategies to heal these wounds is critical to their survival. One such strategy is the formation of a wound periderm that, similarly to native periderm, contains suberized cell walls [1,2]. Suberin is a solvent-intractable heteropolymer found on the inner face of the primary cell wall. Current models describe it as an aliphatic polyester of glycerol and fatty acid derivatives that is crosslinked to polyphenolics and associated with solvent-extractable waxes [3,4,5,6]. It is proposed that the organized deposition of these domains produces the characteristic lamellar pattern observed under transmission electron microscopy (TEM [7]).

Wound responses vary among plant species and organs, and this process has been extensively studied in potato (*Solanum tuberosum* L.). Potato tubers are covered by a suberized periderm, and a suberized wound periderm is formed when this barrier is disrupted, thereby facilitating the study of suberin deposition [8]. Although tuber wound periderm is chemically similar to the corresponding native periderm, it contains lower amounts of suberin and associated waxes than native periderm [9]. In this tuber model, wound suberin deposition occurs in two stages. A closing layer is first formed, where temporary first-response chemicals are deposited, including suberin. This suberization occurs on the cell walls of existing parenchyma cells at the wound site [10]. The completion of the closing layer is followed by the formation of a wound periderm, which is synthesized by a newly created cork cambium (i.e., wound phellogen) that produces multiple layers of suberized phellem cells below the closing layer [10,11]. Precisely controlled temporal and spatial regulation of many genes is required to coordinate the synthesis of polyaliphatics and polyphenolics during the wound-healing process. Although some progress has been achieved [10,12], the molecular mechanisms responsible for the formation of both layers remain unclear.

Most of the research on tree wound responses has been focused on xylem tissue because of its economic value to the forestry sector [13]. The wound-healing mechanism of tree bark has been studied anatomically and histochemically [14,15,16], but almost nothing is known about the chemistry of the biopolymers deposited or the molecular mechanisms underlying this process. Bark, defined as all tissues external to the vascular cambium, contains both living (inner bark) and non-living tissues (outer bark). The inner bark contains the phloem, phellogen (cork cambium) and phelloderm, whereas the non-living component of bark is composed of mature phellem (cork cells [17]). The phellogen, a meristematic layer that produces the periderm, deposits phellem cells in an outward direction and phelloderm inwardly. Phelloderm, phellogen, and phellem compose the periderm, a critical barrier that protects the xylem from fungal and bacterial penetration and prevents water and solute loss (for review, see [18]. The ability of a plant to restore the integrity of the bark periderm upon physical damage is thus critical to its survival. A better understanding of the wound-healing process will allow us to improve this natural barrier to minimize pathogenic microorganism invasion at wounding sites caused by insects, humans, or nature.

Microscopic observations of the chronology of the wound-healing process in tree bark have shown that this process also occurs in two distinct steps, the formation of a ligno-suberized boundary zone followed by the synthesis of a wound periderm [13]. The modifications that take place as a first wound response leading to changes in existing parenchyma cells before the formation of the new wound meristem and periderm form a barrier that has received several names in the literature, such as the closing layer, impervious tissue, and impervious boundary zone [10,12,19,20,21]. Here, we refer to this layer as the ligno-suberized closing layer. The wound periderm displays different histochemical reactions than a natural periderm [22,23]. Within the first 72 h post wounding, lignification occurs in the cells closest to the vascular cambium, with the parenchymatous cells being the first to visibly stain by phloroglucinol/HCl [13]. These lignified cells then become impregnated with suberin 24–48 h after lignification is visible [19,22,24], resulting in a ligno-suberized closing layer found approximately within 4–7 days, and 1 mm from the wound surface. A meristematic layer is generated within 8–9 days post wounding, which establishes the wound periderm within 10 days [13]. As phellem is produced, the ligno-suberized closing layer decreases in thickness [25].

Since the sequencing of poplar (*Populus trichocarpa* [26]), genomic tools have been developed advancing our understanding of the genes and enzymes involved in hardwood tree metabolism, physiology, and stress response. Poplar trees are fast growing and easy to propagate, with many species adapted to a wide range of climatic and soil conditions [27,28]. Here, we report the chemical, anatomical, and molecular characterization of wound-induced suberization in hybrid poplar (*Populus tremula* × *P. alba*). The goals of this study were (1) to determine if genes upregulated during native periderm development are also upregulated in wound periderm formation, and (2) to link the expression of poplar candidate genes potentially involved in the biosynthesis and regulation of this process to the chemical composition of suberin, namely ester-bonded polyaliphatics, crosslinked polyphenolics, and the associated waxes.

## 2. Results

### 2.1. Histochemistry of the Wound-Healing Suberization Process in Poplar Bark

**Figure 1:**
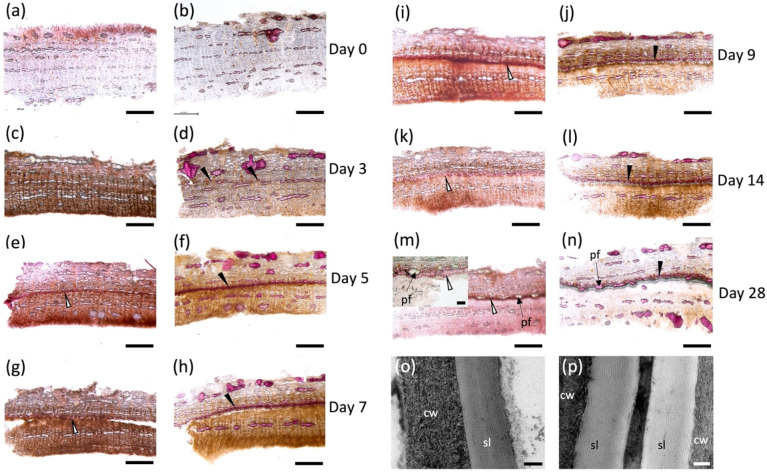
Poplar bark wound periderm development. Cross sections of 7 mm-diameter punches from hybrid poplar bark stained with Sudan red 7B (**a**,**c**,**e**,**g**,**i**,**k**,**m**) and phloroglucinol (**b**,**d**,**f**,**h**,**j**,**i**,**n**). Outside bark in all figures is oriented toward the top. Control tissue sampled at the cut site immediately after wounding (**a**,**b**)**.** Development of wound periderm sampled on day 3 (**c**,**d**), day 5 (**e**,**f**), day 7 (**g**,**h**), day 9 (**i**,**j**), day 14 (**k**,**l**) and day 28 (**m**,**n**) after wounding. Early time points (days 0–4) did not show any evident suberization, but suberized cells were evident by day 5 (**e**) and a well-developed wound periderm is observed on days 9, 14 and 28 (**i**–**n**). (**o**,**p**) TEM images of wound periderm 10 days post wounding (**o**) and native periderm (**p**). cw: cell wall; pf: phloem fibers; sl: suberin lamellae. Black arrow: lignified cell walls; white arrows: suberized cell walls. Scale bars: 500 μM (**a**–**n**), 100 μM for inset shown in (**m**), and 100 nm (**o**,**p**).

Tissue samples excised on each day post wounding (Figure S1a,b) were hand sectioned and stained with Sudan 7B to detect aliphatic suberin deposition [29] (Figure 1a,c,e,g,i,k,m), and phloroglucinol-HCl to detect lignin (Figure 1b,d,f,h,j,i,n). Immediately after wounding, no positive staining was observed with either dye, indicating that the aliphatic and phenolic polymers were absent before the wound-healing process started (Figure 1a,b). Cell walls with positive phloroglucinol-HCl staining were noted as early as day 3 (Figure 1d) located approximately 500–600 μM below the wound surface, but these cells were not suberized at this time point (Figure 1c). Suberized cell walls on the lignified cells were detected on day 5 when a ligno-suberized boundary zone inward to the wound surface was clearly observed (Figure 1e,f); the same cells showed positive staining with both dyes, indicating that the same cell walls co-deposited lignin and suberin. Although Sudan 7B positively stained what appears to be phellem cells at this time, we were unable to localize lamella via TEM. At 7 days post wounding, the ligno-suberized boundary zone did not show any visible changes in staining intensity with either Sudan 7B or phloroglucinol (Figure 1g,h) and seemed to have reached completion. Samples taken 9 days post wounding (Figure 1i,j) showed distinct but adjacent cell layers with positive Sudan 7B (Figure 1i) and phloroglucinol (Figure 1j) reactions, possibly indicating the establishment of a wound meristem and the formation of the new wound periderm deposited internally to the ligno-suberized boundary zone. Suberized cells continued to develop until 14 days post wounding (Figure 1k,l), when a fully formed 60 μM thick wound periderm was observed. A 230 nm suberin lamella was observed by TEM 10 days after wounding (Figure 1p), which is approximately 100 nm thinner than the lamella observed in native periderm of non-wounded tissues (Figure 1o). At 28 days post wounding (Figure 1m,n), a full wound periderm was present with 4–5 rows of newly deposited suberized cells (Figure S1c).

### 2.2. Time Course of Suberin Deposition during Wound Periderm Formation

#### 2.2.1. Suberin-Associated Waxes

**Figure 2:**
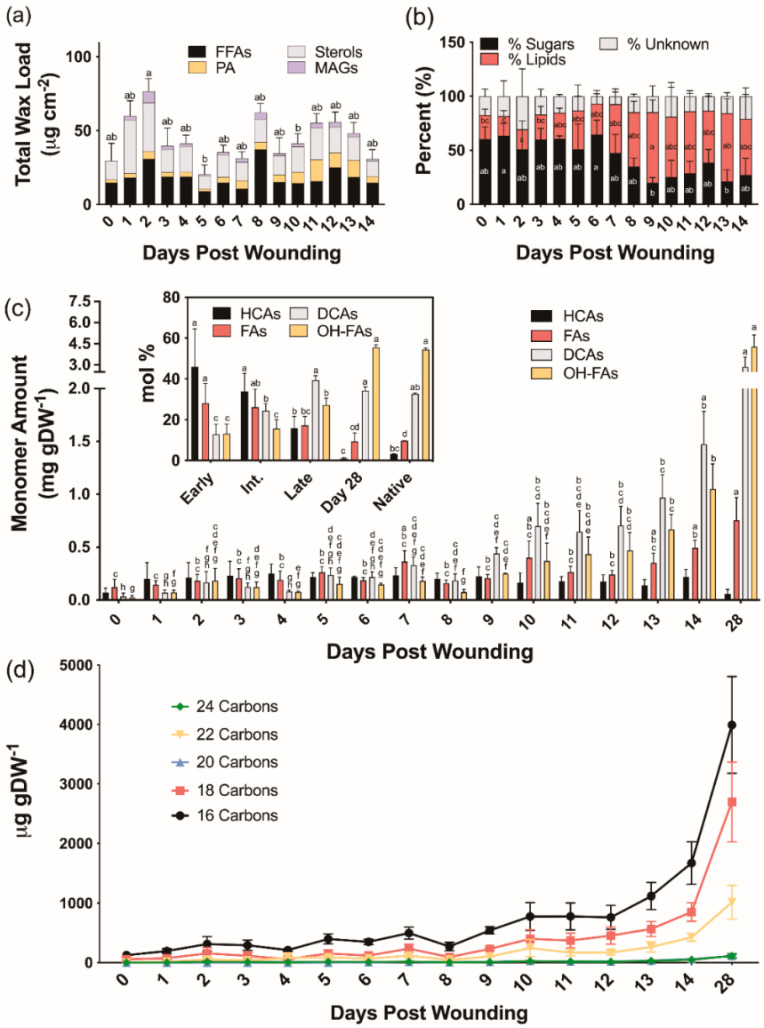
Chemical analysis of aliphatic poplar bark wound periderm development. (**a**) Composition of waxes extracted by rapid chloroform dipping. (**b**) Percentage of quantified components found in the chloroform fraction. (**c**) Aliphatic suberin monomer composition (fatty alcohols were only found in trace amounts 11–14 days after wounding and not shown on this graph). Inset: monomer proportion relative to a fully healed wound periderm and native periderm controls. Means that do not share a letter are significantly different at *p* < 0.05, according to a Tukey post hoc test. Averages ± SD are reported. No such differences were found in the Unknown % (**b**) and HCA data (**c**). (**d**) Aliphatic monomer composition by average chain length of each monomer class of the developing polymer. DCA: dicarboxylic acids; FA: fatty acids; FFA: free fatty acids; HCA: hydroxycinnamic acid derivatives; MAG: monoacylglycerols; OHFA: hydroxy fatty acids; PA: primary alcohols.

We next analyzed extracellular lipid composition in wound-healing poplar bark tissues. The average total chloroform-extracted wax loads were 49.5, 36.8, and 46.5 μg cm^−2^ for early (0–4 days post wounding), intermediate (5–9 days post wounding), and late (10–14 days post wounding) days, respectively (Figure 2a). Unlike the wax profiles observed in native periderm [30], the chloroform-soluble fractions extracted during the process of bark wound healing contained on average 45% sugars, in addition to fatty acids, alcohols, and monoacylglycerols normally found in bark periderm. Both alkanes, which are major components of native bark periderm waxes, and aldehydes, which were quantified as unsaturated TMSi ethers previously, were notably absent in wound-healing tissues (Table S1). Chloroform-soluble lipids extracted from days 0 through 7 post wounding contained 57% more sugar than those isolated from 8 to 14 days after wounding (Figure 2b).

After wax removal, we isolated the remaining soluble lipids by sequential extractions of chloroform, methanol, and isopropanol. Solvent-extracted fractions were pooled and dried under a N_2_ stream, subsequently depolymerized and analyzed by gas chromatography, but only residual fatty acids and sugars were detected (data not shown). Even after separating the lipid extract by thin layer chromatography to remove fatty acids and enrich the preparation in less represented components, neither waxes nor suberin precursors were observed.

#### 2.2.2. Suberin Aliphatic Polyester Monomers

To investigate the composition of ester-bound suberin during the time course of wound healing, cell wall-enriched samples devoid of soluble lipids were depolymerized by base-catalyzed transmethylation. All major monomer classes, namely hydroxycinnamic acids (HCAs), fatty acids (FAs), dicarboxylic acids (α,ω-DCAs) and ω-hydroxy fatty acids (ω-OHFAs), with only traces of primary alcohols, were identified and quantified (Table S2). Total monomer amounts increased from 0.25 ± 0.09 mg g DW^−1^ on day 0 to 7.98 ± 1.09 mg g DW^−1^ on day 28 (Figure 2c). Overall, monomer amounts remained relatively constant until day 9, showing a linear increase thereafter. The monomers released at earlier days (0–4 days post wounding) were mostly HCAs, namely methyl coumarate and methyl ferulate, with values ranging from 41 to 53 mol% of the total monomer load. However, their proportion on days 5–9 post wounding was decreased to 30–45 mol%, and days 10–14 of the wound-healing process had values ranging from 10 to 20 mol%. Between 0 and 4 days post wounding, FAs accounted for 20–38 mol% of the ester-bonded monomers, while later days had a much lower amount (14–24 mol%). As the new wound periderm was established, the amounts of α,ω-DCAs and ω-OHFAs increased sharply from an average of 12.8 mol% to 39.3 mol% and 13.2 mol% to 27.2 mol%, respectively, for the early to late days. Day 28 samples contained 1 mol% HCA, 9 mol% FA, 34 mol% α,ω-DCA, and 56 mol% ω-OHFA which are nearly identical to the 3:10:33:54 mol% breakdown observed for HCAs, FAs, α,ω-DCAs, and ω-OHFAs, respectively, in the native periderm controls (Figure 2c inset). Native periderm samples released a total monomer load of 20.00 ± 1.18 mg g DW^−1^, whereas wound-healing suberin amounts were on average approximately 3%, 5%, and 10% of that for early (0–4), intermediate (5–9), and late (10–14) days, respectively. At 28 days post wounding, likely representing a fully healed periderm, the amount of suberin was only approximately 40% of the native periderm. The chain lengths of the dominant monomers released by transesterification within the first 0–5 days of the wound-healing process were shorter than those identified at later time points. This trend was especially prevalent among the α,ω-DCAs and ω-OHFAs, with longer (i.e., 20–24-carbon) monomers beginning to appear after 9 days post wounding (Figure 2d). In summary, chain lengths were shorter on average in the developing wound-healing polymer than in the mature wound periderm (Figure 2d).

#### 2.2.3. Suberin Glycerol

**Figure 3:**
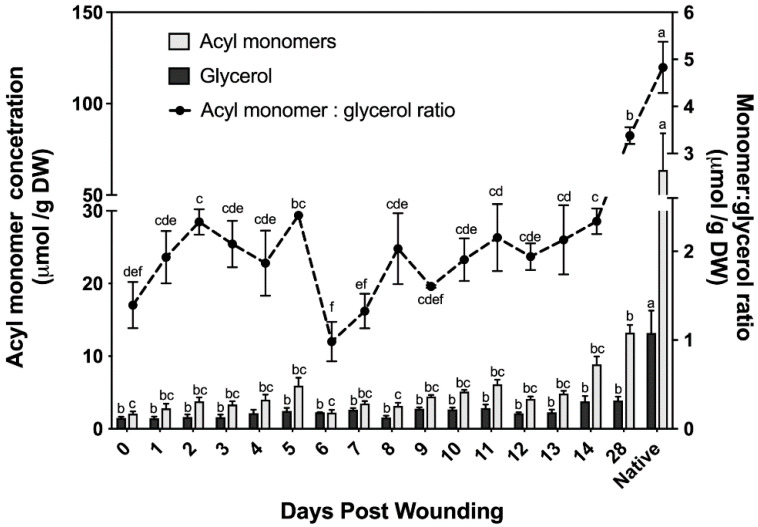
Acyl monomer and glycerol determination of wound-healing suberin including ratio. Acyl monomer composition determined across a 28 day wound-healing period using stable isotope dilution assays for simultaneous determination of glycerol and suberin monomers. Results were compared to a non-wounded native control. All measurements are reported as the average of a minimum of three replicates +/− SE. Means that do not share a letter are significantly different at *p* < 0.05, according to a Tukey post hoc test.

The chemical composition of suberin ester-bound aliphatics detailed above uses organic solvent–aqueous phase partitioning to extract the organic solvent-soluble suberin monomers; glycerol is the only hydrophilic monomer and is discarded with the aqueous phase. Cell wall glycerol content of bark samples was quantified using an isotope dilution assay [31]. This assay allows us to determine the ratio of monomers to glycerol and uses a lower catalyst concentration (the optimized amount for poplar samples was 1% NaOMe; Supplementary Materials, Figure S2) than that used to analyze the suberin data reported in Figure 2 (6% NaOMe). Although similar monomer concentrations to those determined using higher catalyst concentration were found with the glycerol method (Figure 3), this method fails to accurately quantify alcohols; therefore, it was only used to determine the ratio of monomers to glycerol. The amount of ester-bonded glycerol ranged from 1.5 μmol to 3.9 μmol across a 28 day period post wounding. Noteworthy, samples harvested immediately post wounding (i.e., day 0) contained glycerol and ester-bonded acyl monomers, mostly fatty acids.

The monomer to glycerol ratio was found to fluctuate greatly between 0 and 9 days post wounding, before the establishment of a wound periderm (Figure 3). Following the establishment of a new wound periderm, a ratio of 2:1 (monomers:glycerol) was observed 9 to 14 days post wounding. By day 28 this ratio increased to 7:2, which is closer to the 5:1 ratio calculated for non-wounded (native) periderm control.

#### 2.2.4. Polyphenolics Analysis

To analyze polyphenolics from lignin and suberin, we carried out thioacidolysis on the tissue remaining after wax and suberin removal [2]. Thioacidolysis has been routinely used to probe the composition of the polyphenolic domain of suberin; it specifically breaks β-O-4′ ethers providing the relative ratio of each monolignol unit [32,33]. This method uses dioxane, a toxic solvent that forms explosive peroxides over time, is environmentally unfriendly and may cause cancer [34,35]. To address solvent toxicity and stability concerns, we have modified a previously reported microscale thioacidolysis method [36,37], by replacing dioxane with cyclopentylmethyl ether (CPME), a solvent with a high boiling point that is stable under acidic and basic conditions, and has a low rate of peroxide formation [38]. The validation of this modified assay is shown in the Supplementary Materials, Figure S3.

**Figure 4:**
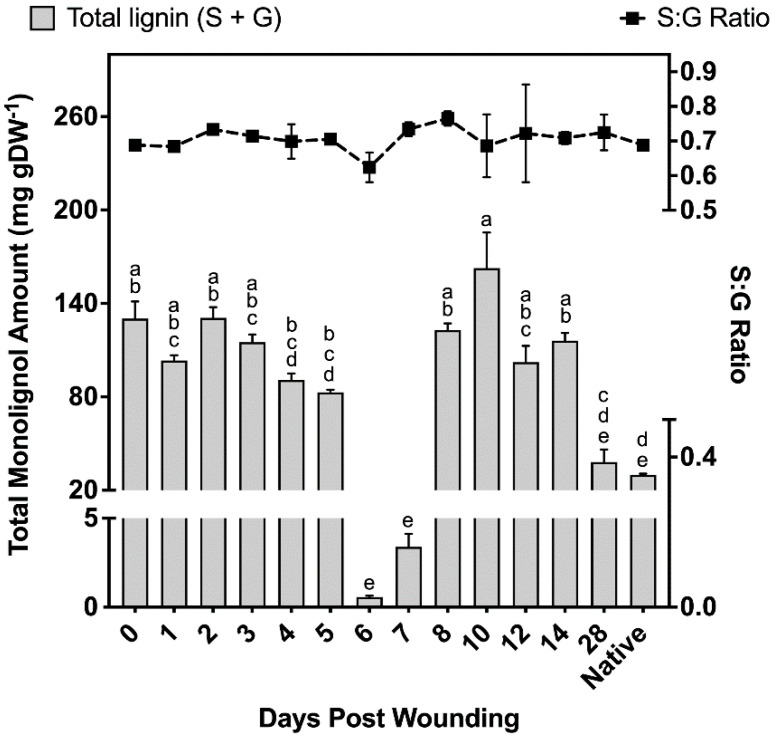
Total amounts of monolignols liberated and the ratio of G:S monomers. Total monolignol amounts released using thioacidolysis, and quantified against the internal standard tetracosane. All measurements are reported as the average of a minimum of three replicates +/− SD. Means that do not share a letter are significantly different at *p* < 0.05, according to a Tukey post hoc test. No such differences were found in the S:G ratio data.

Samples taken at different times after wound healing contained 37–40% S units and 60–63% G units, leading to monolignol ratios of 0.59–0.67 S:G (Figure 4). Non-wounded (native periderm) controls produced similar ratios to those observed in the wound-healing polymer, finding on average 38% S units and 62% G units (Figure 4). However, total monolignol amounts in the non-wounded controls were 75% lower than the observed levels 14 days post wounding. Between days 0 and 5 of the wound-healing process, total monolignol amounts varied between 82 and 131 mg g DW^−1^; however, days 6 and 7 contained significantly lower amounts of monolignol material despite phloroglucinol staining clearly indicating a strong presence of lignin in the completed ligno-suberized boundary zone. These samples produced 0.56 ± 0.09 mg g DW^−1^ and 3.39 ± 0.73 mg g DW^−1^ for 6 and 7 days post wounding, respectively, which corresponds to less than 1% and 4.1% of the monomer amounts quantified 5 days post wounding. On days 8–14, between 102 and 163 mg g DW^−1^ were released, decreasing to 39 mg g DW^−1^ by day 28, an amount not significantly different from that found in natural periderm (Figure 4).

### 2.3. Time Course of Expression of Predicted Genes of Suberin Biosynthesis

**Figure 5:**
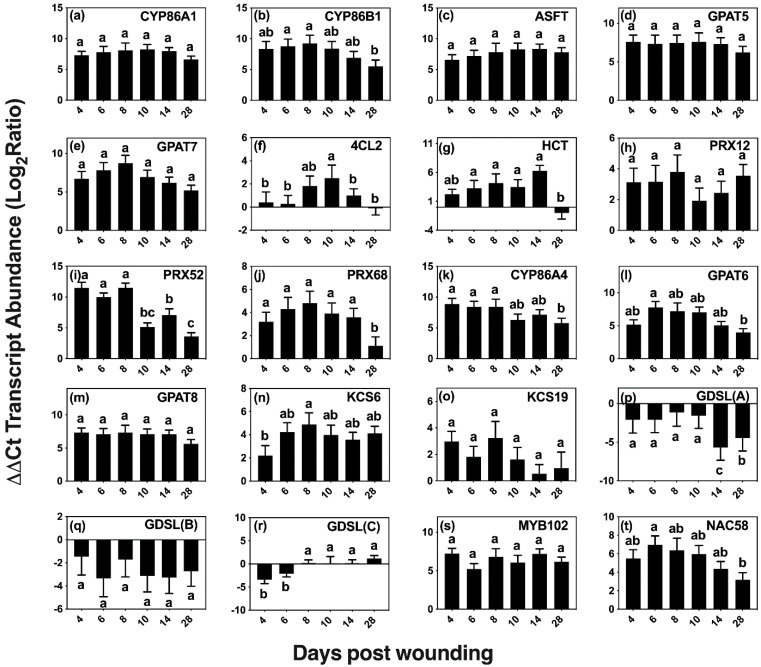
Wound-induced changes in the expression of selected candidate genes of aliphatic and aromatic suberin biosynthesis. Transcript levels of putative genes (**a**–**t**) of aliphatic suberin monomer biosynthesis (*CYP86A1*, *CYP86B1*, *KCS6*, and *KCS19*), phenolic suberin biosynthesis (*4CL2* and *HCT*), ester-formation (*GPAT5*, *GPAT6*, *GPAT7*, *GPAT8*, *ASFT*, and *GDSLs*), phenolic crosslinking (*PRX12*, *PRX52*, and *PRX68*), and regulation (*MYB 102* and *NAC58*) were quantified by reverse transcription-quantitative polymerase chain reaction (RT-qPCR). Gene expression values were normalized to the geometric mean of the endogenous controls *TIP41-like* and *CDC2*. For each transcript, the ratio between normalized gene expression on each day post wounding and day 0 (control) was calculated and log_2_ transformed to facilitate statistical testing. Significance determined using one-way ANOVA, and a Tukey post hoc test, means that do not share a letter are significantly different at *p* < 0.05.

Although wounding triggers a number of responses, we focused on wound-healing suberization, a process that involves acyl-lipid and phenylpropanoid metabolic pathways to form an aliphatic polyester and a phenolic polymer. The expression of 20 candidate genes was quantified via qRT-PCR on days 0 (control), 4, 6, 8, 10, 14, and 28 post wounding (Table 1, Figure 5). Genes were selected using information from the native poplar periderm transcriptome [30], known genes of suberin and cutin metabolism and regulation in other species (including candidate genes from families of key genes involved in these pathways), and genes known to be involved in the wound-healing process.

Two homologs of genes that are involved in monolignol biosynthesis in *Arabidopsis* were included in our analysis, *4-COUMARATE:COA LIGASE 2* (*At4CL2* [39]) and *HYDROXYCINNAMOYL-COENZYME A SHIKIMATE/QUINATE HYDROXYCINNAMOYL TRANSFERASE* (*HCT* [40]). The ligase activity is necessary early in the pathway to form *p*-coumaroyl-CoA, a precursor of ferulate, caffeate and of all three monolignols [41]. The HCT transferase is essential in the pathway that produces ferulate, caffeate, sinapyl alcohol and coniferyl alcohol. Wounding elicited *4CL2* expression at 8, 10 and 14 days after wounding, with a maximum expression (5 fold) observed on day 10.

CLASS III PEROXIDASES are thought to be one of the major enzymes responsible for the oxidative coupling of *p*-hydroxycinnamyl alcohols and *p*-hydroxycinnamates [42,43,44,45]. Homologs of three peroxidase genes shown to be upregulated in the developing phellem, *Potri.005G195700*, *Potri.013G083600*, and *Potri.013G156500* with best hits to *AtPRX12*, *AtPRX52*, and *AtPRX68*, respectively, showed upregulation during wound suberization [30]. *PRX52* (*Potri.013G083600)* relative expression was the highest among the three, with >2000-fold expression up to day 8 and then falling by approximately 50% thereafter. *PRX12 (Potri.005G195700)* presented a similar pattern of expression, but with an up to 11-fold increase over basal levels on days 4–8, whereas *PRX68 (Potri.013G156500)* was consistently upregulated by >15 fold within the first 14 days of wound response and reduced to 2 fold 28 days after wounding.

Homologs of selected genes with confirmed functions in suberin aliphatic metabolism in Arabidopsis and other species showed high upregulation across the 28 day period (Figure 5a–e). These included genes encoding ALIPHATIC SUBERIN FERUOYL TRANSFERASE (ASFT/HHT [46,47]) that links ferulate to ω-hydroxyacids and primary alcohols via ester bonds, the fatty acid omega hydroxylases CYTOCHROME P450 FAMILY 86 SUBFAMILY A POLYPEPTIDE 1 (CYP86A1 [48]) and SUBFAMILY B POLYPEPTIDE 1 (CYP86B1 [49]), and GLYCEROL-3-PHOSPHATE ACYLTRANSFERASE 5 (GPAT5 [50]) and 7 (GPAT7 [51]) that transfer acyl-CoAs to glycerol 3P. Among those involved in aliphatic suberin biosynthesis, *CYP86B1* and *GPAT7* were upregulated by >250 fold on days 4–8 after wounding and steadily decreased after day 8, showing a >30-fold decrease in expression on day 28. The remaining candidates, *ASFT*, *CYP86A1*, and *GPAT5* were consistently upregulated across the time course, with relative expression averages of 120, 250 and 180 fold, respectively.

Homologs of genes involved in the cutin pathway, namely *CYP86A4* [52], *GPAT6* [52], and *GPAT8* [53], which are differentially upregulated in developing bark periderm [30], were also expressed in wound-healing bark (Figure 5k–m). *CYP86A4*, encoding an enzyme involved in the synthesis of 16:0 16-OH FA in Arabidopsis [52], showed high upregulation (>300 fold) on days 4, 6, and 8, correlating well with the observed increases in 16:0 16-OH FA monomers. Expression remained high after day 10; but by day 28, its relative expression level dropped to ca. 60 fold above the basal value.

Two putative *3-KETOACYL-COA SYNTHASE* genes (encoding an enzyme of the fatty acid elongase complex that extends 18-carbon acyl chains), homologs of *KCS6* [54] and *KCS19*, were analyzed because of their high expression in poplar bark phellem (Figure 5n,o). Whereas *KCS6* presented a maximum expression (ca 32 fold) on day 8 and was upregulated throughout the wound-healing process, *KCS19* showed much lower levels of upregulation with the highest expression value (ca. 8 fold) found on day 8.

We also analyzed the expression of three putative *GDSL-MOTIF ESTERASE/ACYLTRANSFERASE/LIPASE* family genes, including a homolog of *CUTIN SYNTHASE 3* (*CUS3*) that is among the 50 most upregulated genes in bark phellem [30]. Both CUT3 and CUT4 are closely related to CUS1 [55], the enzyme that assembles the cutin polyester extracellularly [56,57]. We also included an additional *GDSL* that highly represented in the poplar phellem transcriptome. However, all three were downregulated after wounding (Figure 5p,r).

We explored the expression of two putative transcriptional regulators, *MYB102* and *NAC58*, that are upregulated in the native poplar periderm, and also showed high expression during the wound-healing process (Figure 5s,t). The Arabidopsis MYB102 transcription factor, which belongs to the same clade as MYB41, an activator of suberin biosynthesis under abiotic stress [58], has been shown to be induced by osmotic stress, wounding, ABA, and jasmonic acid [59]. Similarly, StMYB102 regulates wound suberin biosynthesis in potato tubers [60]. The *AtMYB102* homolog, Potri.011G041600, showed >60-fold increase during the time course of wound-healing suberization without showing significant expression changes among the days analyzed. The second transcriptional regulator investigated, *NAC58*, belongs to a largely uncharacterized family believed to play a role in plant development and stress response [61]. A potato homolog to AtNAC58, StNAC103, has been shown to repress suberin deposition [62]. Its closest poplar homolog gene, Potri.015G046800, showed consistent upregulation, with near uniform values across the first 10 days post wounding, followed by a decrease in expression on days 14 and 28.

## 3. Discussion

Suberin biosynthesis is a coordinated production and co-localization of two chemically distinct domains: polyaliphatic and polyphenolic. Chemical compositions of aliphatic suberin have been reported for several species including hybrid poplar [30] and can be used in conjunction with genomic data to deduce important biochemical events and generate new hypotheses. Several genes encoding enzymes required for suberization have been discovered and functionally characterized in *Arabidopsis thaliana* and potato. C16–C18 acyl chains generated in the plastid are transported to the ER membrane for further modifications [48,49], producing suberin precursors. These modifications include elongation, oxidation, reduction, and transfer steps before being assembled into polymeric suberin (reviewed by [63], and involve several families of enzymes, including CYP86 subfamily of P450 monooxygenases, long-chain acyl-CoA synthetases (LACS), glycerol-3-phosphate acyltransferases (GPAT), fatty acyl reductases (FAR), and components of the fatty acid elongation (FAE) complex. Additionally, hydroxycinnamic acids are esterified to omega-hydroxy acids and alcohols by HXXXD-motif transferases [46,64,65]. In part, these remain ester bound to suberin polyaliphatics, but another fraction is crosslinked into the polyphenolic suberin polymer [3], which is believed to be synthesized by a similar pathway to that of lignin.

Mechanical injury of tree bark induces the formation of a suberin-enriched wound periderm, a process that has been described in wound-healing potato [9,10,11,12,66,67]. However, it is unclear to what extent conclusions made on the potato model can be transferred to the formation of wound periderm in woody angiosperm bark. We have investigated the time course of wound suberization in poplar, which has allowed us to closely relate the expression of candidate genes for poplar suberin metabolism [30] with the chemical composition of developing cell wall-associated polymers.

### 3.1. Microscopic Analysis of Bark Wound-Healing Suberization

The histochemical analysis of suberin and lignin deposition during the time course of wound response followed the sequence of events observed for this process in other species. The location and timing of formation of the closing layer agrees with previous reports in woody angiosperms, showing that suberization occurs 48 hrs after deposition of lignin in the cells several layers below the wound surface (Figure 1c–f [13,22,25,68]). Although suberin-specific monomers were found by chemical depolymerizations as early as day 2, Sudan 7B did not clearly indicate the presence of suberin until 5 days post wounding when the lignified cells of the ligno-suberized closing layer became impregnated with suberin. The monomers released early in the wound-healing process could be the result of incipient suberization of the closing layer not detected histochemically by Sudan 7B.

Both the histochemistry and lipid analyses suggest that a wound periderm was only formed 9 days after wounding. Likewise, a new meristem is formed internal to the ligno-suberized closing layer in the bark of peach tree 8–9 days after wounding [13]. Importantly, although cells in the closing layer exhibited both positive sudan 7B and phloroglucinol-HCl staining, cells present in the wound periderm show a clear distinction between suberized and lignified cells (Figure 1m,n). These differences in histochemical staining suggest that the polyphenolic domain of wound suberin is chemically different from that of lignin formed in the closing layer. TEM samples prepared from tissues 5 days after wounding did not show suberin lamellae in the cell wall, and similar to peach bark [22], the lamellae were not detected until day 10 (Figure 1o). Pronounced differences in monomer proportions during early and intermediate wound healing (Figure 2c), including low monomer:glycerol ratio (Figure 3), may explain the lack of lamellae when these tissues were observed by TEM. A well-formed wound periderm was observed 9 days post wounding (Figure 1i,j) with complete formation after 28 days (Figure 1m,n), albeit the total suberin amount in tissue harvested 28 days after wounding is only 40% of the amount found in native periderm. Similarly, potato wound periderm contains only 50–60% of the wax and suberin loads of the native periderm [9].

### 3.2. Polyphenolic Suberin Biosynthesis and Composition

It is thought that suberization starts with the biosynthesis of the polyphenolic domain, followed by that of the polyaliphatic domain [69]. Therefore, it is expected that expression of genes involved in the phenylpropanoid pathway and the synthesis of their corresponding chemical products will occur immediately preceding the deposition of aliphatic suberin. The polyphenolic domain of suberin was analyzed by thioacidolysis, a method routinely applied to study lignin monomer composition that cleaves β-O-4 ethers of monolignols to liberate H, G, or S thioethylated monomers [33]. Consequently, when thioacidolysis is applied to suberized tissues, it is not possible to chemically discriminate between monolignols derived from lignin and suberin polyphenolics. We found a S:G monomer ratio of 40:60, which was consistent across the 14 day period examined and agrees with the ratio reported for potato wound and natural periderms [2,33]. Studies performed in poplar have found S:G ratios of 48:52 in unwounded bark tissues [70], and ratios of 60:40 in debarked wood samples [33,71]. Our non-wounded bark controls show ratios of 40:60 (S:G), which are consistent with wound-healing ratios determined, illustrating that the ratio of monolignols is not identical among poplar species and could be influenced by environmental conditions [72,73]. Differences in monolignol amounts and S:G ratio between suberized periderm and poplar wood suggest that this polymer is compositionally different in these tissues. However, although thioacidolysis releases β-O-4-etherified ferulic acid from lignin [74], we have not identified even traces of this monomer in our GC–MS analysis of thioacidolysis products. Because a high amount of background lignin is present in poplar bark tissues, high concentrations of monolignols are released from samples harvested immediately after wounding. As a result, any small increases in monolignols from suberin polyphenolics present in the ligno-suberized boundary zone and the newly deposited wound periderm may be masked. Clearly, lignin deposition during the formation of the ligno-suberized boundary zone could be detected by phloroglucinol 3 days after wounding, increasing in intensity over time up to day 7, but such increase was not reflected in the amounts of lignin monomers released by thioacidolysis, remaining either similar to the control tissue or containing lower concentrations than the control. Our results suggest that significant cell wall remodeling or tissue necrosis occurs 6–7 days post wounding, when the monolignol amounts were observed to drop to less than 4% of the amounts found in the preceding days, coinciding with the completion of the ligno-suberized boundary zone formation. It is possible that cell wall remodeling renders the monolignols insoluble to the thioacidolysis process, since the ligno-suberized boundary zone clearly reacted positively to phloroglucinol-HCl on days 6–7 after wounding (Figure 1g,h). Between 8 and 14 days after wounding, the concentrations of monolignols measured by thioacidolysis were similar to the initial levels, but dropped substantially by the time the wound periderm was completed (day 28), reaching a similar value to that of native periderm.

Peroxidases have been implicated in phenolic domain polymerization [43,44,45] and monolignol crosslinking by catalyzing the oxidative coupling forming β-O-4-ether linkages in lignin biosynthesis [75,76]. AtPER64 has been shown to be required for polyphenolic assembly in *Arabidopsis* casparian strip formation [77], and AtPER3, 9, 39 and 72 are also implicated in this process [78]. We investigated three peroxidases that are upregulated in the native poplar bark transcriptome [30]. The expression pattern of *PRX52* (Figure 5i) correlates with the generation of a ligno-suberized boundary zone and regeneration of wound phellogen. Furthermore, this pattern of expression mirrors that found for other suberin related genes in potato, which was attributed to completion of the ligno-suberized closing layer followed by meristem regeneration [10]. Each of the three peroxidases investigated here remained upregulated 28 days post wounding, supporting the observation that oxidative crosslinking of the phenolic domain may be controlled by multiple peroxidases [10,44]. The expression patterns of *HCT* and 4 *CL2* across all days investigated indicates that these isoforms are required for the synthesis of phenylpropanoids throughout the formation of the closing layer and wound periderm (Figure 5f,g). Coumarate and ferulate are normally esterified to suberin aliphatics; however, the increase in these monomers observed as an immediate response to wounding does not parallel the accumulation of polyaliphatics that occurs later during the formation of the closing layer.

### 3.3. Aliphatic Suberin Biosynthesis and Composition

Previous studies have found similar compositions of wound periderm suberin relative to native controls and identified more noticeable differences in the wax fraction [9]. Our study also identified remarkable differences in the composition of waxes extracted from the wound periderm, dominated by free fatty acids, alicyclics and alcohols, compared to those found in poplar bark periderm, composed mainly of fatty acids, primary alcohols, and alkanes [30]. In addition, wound suberin, particularly in the relative proportion of ester-bound HCAs across the first 14 days of wound healing (Figure 2c). Although native bark periderm control tissues contained on average 2 mol% ester-bound HCAs, wounded tissues contained between 10 mol% on day 13 and 53 mol% on day 4. Transcripts of *ASFT* homolog, which encodes a feruloyl-CoA transferase in other species [46,47,79], remained highly abundant throughout the entire wound-healing process. Contrastingly, the expression of its homolog in wound-healing potato periderm, *FHT*, showed two expression peaks, which the authors attributed to the gene participating in the tightly controlled regulation of ferulate esterification in the ligno-suberized boundary zone and wound periderm formation [10]. In poplar, coumaric acid is the dominant monomer of the HCA fraction released by transesterification of the developing wound periderm and the gene responsible for its incorporation has yet to be identified. Because methanolysis breaks ester bonds and is not specific to those contained in suberin, we cannot attribute released HCAs exclusively to the wound periderm. For example, HCAs could be also attached to the cell wall polysaccharides as shown in grasses [80]. The high content of HCAs in the healing tissue could be the result of a HCA enriched polymer being deposited during the formation of the closing layer, and further crosslinking of these monomers to the cell wall could explain why the percentage of these monomers decreases with wound periderm maturation (Figure 2c, inset). However, feruloyl- and coumaroyl-esters attached to cell walls could be found as an initial response to the injury. Given that HCAs (ca. 70 µg/g DW) are found in control tissues (day 0), these HCAs are possibly already esterified to the cell walls before suberization takes place.

Wound-healing tissues also contain different proportions of FAs and ω-OHFAs than native periderm suberin. However, by day 28, the relative amounts of all major monomer classes are similar to those in native periderm tissues (Figure 2c inset). Very long chain monomers, particularly those with 22 carbons, showed an increase from day 8 onwards. A similar pattern was observed in the waxes with the relative proportion of 24 and 26 carbon alcohols increasing over time; however, the dominant fatty acid components were 16 and 18 carbons in length. In agreement with the monomer chain lengths increasing in the developing polymer, the poplar homolog to *KCS6*—encoding a critical enzyme for the elongation of fatty acid precursors in potato periderm formation [81]—showed consistently upregulated expression as suggested by transcript analysis. The expression of *KCS6* peaked on day 8, immediately preceding the observed increases in average chain lengths of the alcohols in the wax fraction (Table S1) and of ω-OHFAs in the aliphatic suberin polyester (Figure 2d; Table S2) and. *KCS19*, which is upregulated in outer bark tissues under normal development [30], showed no significant differences in expression over time, suggesting it may not play a major role in wound periderm formation.

To infer possible monomer connectivities during the polyester development, glycerol concentration was measured in the time course of wound response. Although solvent-extractable membrane lipids were thoroughly removed before glycerol determination, a relatively high basal glycerol amount was found immediately after wounding (day 0), in addition to FAs and HCAs. It could be postulated that it derives from residual membrane lipids, but control assays applying this depolymerization method to phospholipids and galactolipids have shown that these polar lipids do not contribute to the pool of quantified glycerol [31]. Thus, even if solvent-soluble (i.e., membrane) lipids were not completely removed from our samples, the glycerol released from samples taken before the wound-healing process started is unlikely released from residual membranes. Overall, the concentrations of glycerol and acyl monomers appear to follow a linear trend increasing until day 5, followed by a 60% decrease in the concentration of acyl monomers and, consequently, of the monomer to glycerol ratio. This change in ester-bound aliphatics coincides with the drastic drop in phenolics observed on days 6 and 7 (Figure 4). The low acyl-monomer:glycerol ratio (2:1) across the first 5 days of the healing process, when the proportion of fatty acids (end points of the polymer) is high, suggest that only discrete oligomers are formed during the development of the closing layer. A monomer:glycerol ratio of 2:1 was also calculated for days 9–14 after wounding, however, the acyl composition is higher in ω-OHFAs and α,ω-DCAs compared to that of the early days after wounding, allowing for extended oligomers/polymers to be produced. These structural arrangements may be crucial to form the lamellae observed by TEM (Figure 1o,p).

In agreement with the accumulation of functionalized fatty acids, the ω-hydroxylase transcripts, *CYP86A1*, *CYP86B1*, and *CYP86A4* were consistently upregulated across the wound-healing period, although CYP86A4 is a cutin-specific hydroxylase in *Arabidopsis* [52]. By day 28, the monomer:glycerol ratio increased to 3.5:1, which differs from the 5:1 ratio found in natural poplar phellem cells but is similar to the ratio of 3.7:1 calculated from an earlier report on wound-healing potato suberization [82]. Additionally, consistent with the chemical data is the fact that transcripts of *GPAT5*, *GPAT6*, *GPAT8* and *GPAT7* homologs, were also upregulated across the first 14 days post wounding. Although GPAT6 and GPAT8 are cutin-specific in *Arabidopsis* [52,53], the poplar homologs might have a role in suberization, given the results found in this study (Figure 5) and in our analysis of the native phellem transcriptome (Rains et al., 2018). Unlike potato, in poplar *GPAT5* remained upregulated throughout the wound-healing process [12]. Glycerol content of the polymer increases as a function of time up to day 14, correlating well with upregulation in *GPAT* transcripts. Unlike the other aliphatic monomers, glycerol did not increase between days 14 and 28, although the amount of monomers liberated increased. This would be consistent with a GDSL-like suberin polyester synthase that transfers the acyl group of monoacylglycerol to the growing suberin polyester, releasing glycerol as a sub-product [56]. In this scenario, an elongating polymer would not incorporate acyl monomers in the same proportion as glycerol. At the time these experiments were conducted, a GDSL-family protein with suberin synthase function had not yet been identified, and the three GDSL genes that were highly upregulated in the native poplar phellem transcriptome were analyzed here were largely downregulated in wound-healing samples (Figure 5). Thus, it is possible that, unlike most of the enzymes included in this study which may be common to both native and wound suberin biosynthesis, specific GDSL enzymes may be involved in wound response suberin catenation.

*MYB102* and *NAC58*, encoding transcriptional regulators that belong to families with members involved in stress response and suberin regulation [58,59,60,62,83], were strongly expressed throughout the healing process. Given its pattern of expression, *MYB102* could function in suberin deposition across the whole wound-healing process, including the closing layer and wound phellem suberization. This is an interesting observation, given that *MYB102* is not upregulated in tissues undergoing natural suberization, such as potato skin periderm formation [84], but StMYB102 is one of the transcriptional regulators of wound suberization in potato tubers [60]. Furthermore, *MYB102* is upregulated in surface tissues of russeting apples, which develop a wound-healing periderm [85]. A homolog to NAC58 in potato, AtNAC103, was previously shown to repress suberin deposition [62]. In our study, *NAC58* remained highly upregulated throughout the wound-healing process. The high expression of these two transcription factors during wound healing may indicate a possible role in regulating wound-induced suberin deposition, and suggest that both positive and negative regulators of suberin biosynthesis may modulate the spatio-temporal accumulation of this highly organized polymer.

## 4. Materials and Methods

### 4.1. Plant Materials

Five-year-old *P. tremula* × *P. alba* hybrid poplar clone INRA 717-1B4 trees were grown at the Ontario Forest Research Institute in Sault Ste. Marie, Ontario in pots. Trees were placed outside during the growing season (May–October), and supplemented with fertilizer (20:20:20) once per week.

### 4.2. Bark Wounding

Following the methodology described by Biggs and coworkers (Biggs 1985b; Biggs 1996), stage IV tissues (as defined in [30]) on four 5-year old hybrid poplar trees were wounded with a 7 mm cork borer in a semi-spiral line (Figure S1), spaced evenly around the diameter of the tree to a depth no greater than 1⁄2 the bark thickness (approx. 1 mm). All trees were inflicted with 30 wounds, avoiding overlapping of wounds on the trunk of the tree (i.e., only one wound in any given vertical or horizontal line). The same 7 mm borer was used to remove the wound-healing tissue samples from each tree, sampled to a depth of the xylem at the initial wound site, at 0 (control), 1, 2, 3, 4, 5, 6, 7, 8, 9, 10, 11, 12, 13, 14, and 28 days after wounding. Samples of three biological replicates for RNA extraction and chemical analyses were immediately placed in liquid nitrogen after harvesting and subsequently stored at −80 °C until use. An additional sample from each tree was harvested and cut in half for TEM and histochemical analysis. Samples for TEM were sectioned to produce approximately 1 mm^2^ pieces and fixed immediately, and samples for light microscopy were sectioned by hand for subsequent microscopical analysis as outlined below.

### 4.3. RNA Extraction and cDNA Synthesis

Tissues were harvested 0, 4, 6, 8, 10, 14, and 28 days post wounding from three trees to provide three biological replicates, following the method explained above, and ground in liquid nitrogen. Total RNA was extracted from each powdered wound-healing tissue using the Spectrum^TM^ Plant Total RNA Kit (Sigma Aldrich, St. Louis, MO, USA) according to the manufacturer’s instructions and stored at −80 °C until use. The RNA was DNase treated (Turbo DNase, Invitrogen, Waltham, MA, USA) following the manufacturer’s instructions. RNA purity was assessed using 1% agarose gel electrophoresis and OD_260/280_ and OD_260/230_ ratios using a NanoDrop ND-1000 UV-VIS spectrophotometer (Thermo Fisher Scientific, Inc., Ottawa, ON, Canada). Subsequent synthesis of cDNA was carried out using Maxima^®^ First Strand cDNA Synthesis kit for qPCR (Thermo Fisher Scientific Inc., Ottawa, ON, Canada).

### 4.4. Quantitative Real-Time Polymerase Chain Reaction (qRT-PCR)

The transcript levels of 20 genes were quantified using the comparative cycle threshold (Ct) method (ΔΔCt method [86,87]), with adjusted efficiencies, as described previously [30,88]. All efficiencies were determined using the relative standard curve method with three replicates and endogenous controls *TIP41-LIKE FAMILY PROTEIN* (*TIP41*) and *CELL DIVISION CONTROL 2* (*CDC2*), which have been previously validated for use as reference genes for qRT-PCR analysis [30]. Efficiencies were determined from the slope of the relative standard curve linear regression as described by [86], where E = 10 (−1/slope) and the percent efficiency is E% = (E−1) × 100. Primers for select poplar genes (Table 1) were designed based on the genome sequence of hybrid poplar v2.0, available from the AspenDB at aspendb.uga.edu [89] using Primer3web version 4.1.0 [90,91]. A minimum of three technical replicates for each of the three biological replicates were carried out for each sample using PowerUp™ SYBR™ Green Master Mix (Applied Biosystems, Waltham, MA, USA) for real-time detection of the resulting amplicons using a StepOnePlus (Applied Biosystems) instrument. Ratios are expressed as a function of the mean of day 0 to the mean of each day post wounding relative to the transcript abundance of the geometric mean of the above endogenous controls, and log_2_ transformed to facilitate statistical testing. One-way analysis of variance (ANOVA) was performed for each gene over the time series with a Tukey HSD post hoc test and deemed significant if *p* < 0.05.

### 4.5. Total Lipids and Wax Analysis

Three biological replicates each were ground in liquid nitrogen, and waxes were extracted by 1 min shaking in 5 mL of chloroform. The extracts were moved to clean tubes and 10 μg of the internal standards *n*-octacosane, 1-pentadecanol, and heptadecanoic acid were added. Samples were dried under a stream of nitrogen, trimethylsilyl (TMSi) derivatized, and analyzed via gas chromatography–mass spectrometry (GC–MS) to determine peak identity and quantity, according to the method used by [30]. Tissues remaining after wax removal were subjected to sequential solvent extractions to completely remove soluble lipids, as described by [92]. Pooled organic fractions were dried under nitrogen and TMSi derivatized prior to GC–MS analysis.

### 4.6. Lipid Polyester Analysis

Dry delipidated residues (ca. 5 mg) enriched in cell walls and internal standards, ω-pentadecanolactone and methylheptadecanoate, were depolymerized by methanolysis in the presence of sodium methoxide, which releases fatty acids as methyl esters, following the method described in [30].

### 4.7. Glycerol Quantification

Tissues from days 0–14 and 28 days post wounding were subjected to sequential solvent extractions and depolymerization, as described by [31] with some modifications. After exhaustive delipidation, 5 mg samples were depolymerized with methanol and 1% (*w*/*v*) sodium methoxide catalyst, the minimum concentration found to ensure complete depolymerization (Figure S2). Internal standards added to each reaction (10 μg each) were ω-pentadecanolactone, methyl heptadecanoate, and [^13^C_3_] glycerol (BOC Sciences, Shirley, NY, USA). The released monomers, including glycerol, were acetylated and quantified by total ion current via GC–MS in both the full scan and the SIM mode as reported [31]. Aliphatic monomers quantified using this protocol were used for the sole purpose to determine the monomer to glycerol ratio.

### 4.8. Glycerol Determination Using a Commercial Kit

Following depolymerization isolation of the monomer products according to Yang (2016), dried residues were suspended in 1 mL sterile water and filtered through glass wool. The particulate was washed with a further 200 μL of sterile water and the filtrates were combined. Samples were concentrated by lyophilizing and suspended in 70 μL sterile water prior to glycerol analysis using the commercially available MAK117 kit (Sigma Aldrich, St. Louis, MO, USA). Protocol was followed according to manufacturer’s instructions, and glycerol amounts were calculated based on a colorimetric assay (Figure S2d).

### 4.9. Polyphenolic Analysis: Modified Microscale Green Solvent Thioacidolysis

After suberin removal, 5 mg of the remaining material was rinsed thoroughly with methanol and water to remove traces of sodium methoxide and methyl acetate, followed by an enzymatic cell wall isolation as reported by [36]. A modified thioacidolysis procedure based on previous approaches [36,37] was developed. Approximately 2 mg of cell wall-enriched tissue was weighed, 10 μG of tetracosane added as an internal standard, and the tissue was suspended in 1 mL 2.5% BF_3_ and 10% ethanethiol in dioxane or cyclopentylmethyl ether under nitrogen. This mixture was heated at 100 °C for 4 h with shaking every h. The reaction was cooled on ice for 5 min and terminated by the addition of 0.4 M sodium bicarbonate on ice until the reaction was neutralized. After adding 1 mL water, the mixture was extracted twice with 0.5 mL ethyl acetate. The combined organic fraction was then dried under nitrogen, and the released monomers were derivatized into TMSi adducts and quantified via GC–MS using tetracosane as an internal standard. As a control, the original thioacidolysis method using dioxane [36,37] was applied to control poplar wood, poplar stage III periderm, potato periderm (Figure S3). Correction factors previously determined by [71] for GC–MS quantification of thioacidolysis products using a tetracosane internal standard were applied, namely 0.47 and 0.53, for the G and S monomers, respectively. All amounts were quantified based on total ion current and a minimum of three biological replicates. One-way analysis of variance (ANOVA) was performed over the time series with a Tukey HSD post hoc test and deemed significant if *p* < 0.05.

### 4.10. Microscopy

Transverse sections were obtained using a double-edged razor blade on each day post wounding. Sections were stained in a 1 mg/mL solution of Sudan 7B in 95% (*v*/*v*) ethanol for 20 s at 70 °C, thoroughly rinsed with water and mounted in 50% (*v*/*v*) glycerol prior to imaging. Phloroglucinol-HCl was prepared as a 2% (*w*/*v*) solution in 95% ethanol with 1 volume of 18% HCl [93], and sections were stained for 5 min before rinsing in water and mounting in 50% (*v*/*v*) glycerol. All images were acquired with a Leica M205 FA microscope (Leica Microsystems Inc., Toronto, ON, Canada). For transmission electron microscopy, tissues of approximately 1 mm^2^ were fixed immediately after harvesting in a 4% (*v*/*v*) solution of glutaraldehyde in 0.07 M phosphate buffer, pH 6.8, for 12 h at 4 °C. Fixed tissues were rinsed in buffer until no glutaraldehyde odor was detected, and further sample processing and TEM imaging were performed at the Michigan State University Center for Advanced Microscopy, following the procedures described by [46].

## 5. Conclusions

Although the anatomy and histology of wound healing in stems of tree species has been studied in detail, these studies did not investigate such process at the chemical and molecular levels. A better understanding of the development of cell wall-specific lipid barriers during bark wound response in poplar is crucial to implement strategies to avoid or minimize wounds and the subsequent infections that occur at these sites. This study represents a comprehensive analysis of the developing polymer, encompassing histochemical changes, chemical composition, and candidate gene expression across the time course of wound-healing suberization of poplar tree bark. By fully characterizing the chemical composition of the developing aliphatic domain, including a quantitative analysis of glycerol, we found that the proportions of aliphatic monomer classes as well as the monomer to glycerol ratio are different during the initial stages of suberization compared to that in the final product. These results combined with ultrastructural observations suggest that the polyester formed during the initial stages of wound-healing suberization contains high proportions of FA, HCA and glycerol, and such monomer balance may not be suitable to assemble into the lamellar structure typically observed by TEM. Finally, this study shows that homologs of selected candidate genes known to be involved in native suberin and cutin biosynthesis in other species, and which are upregulated during suberization of native poplar bark periderm, are highly upregulated in wound suberization, suggesting redundant roles for these in suberization during normal development and upon mechanical stress. The candidate genes studied here and the revealed changes in chemical composition of suberin across the wound-healing process provide the foundation for future studies aimed to understand this complex and important process in trees.

## Figures and Tables

**Table 1:** Primers used for qRT-PCR quantification of gene expression of target genes for suberin biosynthesis.

Poplar ID	Best TAIR 10 BLAST	Annotation	Left Primer	Right Primer
Potri.001G036900	AT3G21240	4CL2	ATTCATCAGGGACTACAGGGTT	GCAACACACACAAAATCACATCT
Potri.014G166600	AT5G41040	ASFT	TGAAACAAGGAAAGCCAACTCT	CCTCAACAAAAACAGCACCTTC
Potri.004G133500	AT3G48750	CDC2	TTACTTCTTTGCCCGACTTCA	TATTCATGCTCCAAAGCACTCC
Potri.009G043700	AT5G58860	CYP86A1	GCGTTTATATCCGTCAGTACCACAA	GCTCTTCATCCGTCCAACAGAATAA
Potri.014G085800	AT1G01600	CYP86A4	CTTTCCTTCAACACGTAGCACTCAA	TTGATCAGGACCGTGCAAATTTCAT
Potri.005G092200	AT5G23190	CYP86B1	GCAGGAGATAAAGAAGATGGACTA	TATTGCCTCCATCCTTCCCA
Potri.019G024800	AT5G18430	GDSL (A)/CUS3	TATTGTCAAACTTGTGCCCTAACCG	ATGATGGTGCTGAGGTTCATAGGAT
Potri.001G191400	AT3G16370	GDSL (B)	AGATGCTCTGGTTCCTGCTATCATA	GGGAGGGTAATTGGCCTTGAATATC
Potri.002G253400	AT4G28780	GDSL (C)/CUS4	TGCTTTCTTTGTGTTTGGTGACTCA	GGGAAGTTGAAGCCATTAGAGAAGC
Potri.010G201200	AT3G11430	GPAT5	TGGCAATGAATTACAGGGTTGGTTT	GGCAACTGATTCAAGAAAGTCACCT
Potri.008G058200	AT5G06090	GPAT7	TTGAGTCAGTTGCCAGTAGAAGCTA	TCGTGCATTCAAATCCTAACGTAGC
Potri.014G085500	AT4G00400	GPAT8	CCTTGATCGCTTGCCAGAAGAAATA	TCGAATCCTAACACTTCACCCAAC
Potri.018G105500	AT5G48930	HCT	AGAGAGACACCCAACAGGAC	TAGAACGGCACAAGAACATCAC
Potri.006G218000	AT2G26250	KCS10	TGTCACGCCCAAGGTCTATTTATCT	ATCAAACTTGCCTGATTTCCTTGCT
Potri.013G119600	AT5G04530	KCS19	TAGAGGGAAGAGAGGAATCTGCAAC	AGAACAGAGACACCGAAGAGACAAT
Potri.010G125300	AT1G68530	KCS6	AACACCTCATCGTCCTCGCTTT	CAAACCCTGTCTCCTCTCCTCATC
Potri.011G041600	AT4G21440	MYB 102	TCCAATAACAGCAACCAGACCTTTG	TAGCTATCCCTCTCATCTTCAGTGC
Potri.015G046800	AT3G18400	NAC 58	AACCATCCTCTCAGCAATCTCTAGG	CCACTTGATGAACTGGCAATACGAT
Potri.005G195700	AT1G71695	PRX12	TTACCTCAGACCAAGACCTGTACTC	CTGACAATACGCTAAGCTGTGACAT
Potri.013G083600	AT5G05340	PRX52	CCACCTCCAACTTCTAACTTGAACC	ATATATGCGTGCCCTAAAGTTCGTG
Potri.013G156500	AT5G58400	PRX68	ACTCCTCAGCTCAACTCTCAACAAA	AAAGAACAAGCGAACAAGAGAAGCA
Potri.009G093200	AT4G34270	TIP41 like	TAACTGGCTGGAAACAAGAGG	TTTCACTACCACAATAAGGCGT

## Data Availability

Data are contained within this article or Supplementary Materials.

## Supplementary Materials

The following supporting information can be downloaded at: https://www.mdpi.com/article/10.3390/plants11091143/s1,: Figure S1: Wounding approach and diagram of wound periderm formation in poplar bark; Table S1: Wax components extracted by chloroform immersion on days 0–14 post wounding; Table S2: Suberin monomer concentration in wound-healing bark on days 0–14 post wounding; Text: Optimization of a stable isotope dilution assay for glycerol quantification in poplar periderm suberin; Figure S2: Optimization of a stable isotope dilution assay for glycerol quantification in poplar periderm suberin; Text: Modified microscale green solvent thioacidolysis assay; Figure S3: Modified thioacidolysis method validation. References [94,95,96,97,98,99,100,101,102,103] are cited in the supplementary materials.
