# Supplementary material for: Chemical and Molecular Characterization of Wound-Induced Suberization in Poplar (Populus alba × P. tremula) Stem Bark
Source: Plants (Basel). 2022 Apr 22;11(9):1143. doi: 10.3390/plants11091143 (PMC9102228; doi:10.3390/plants11091143)
Supplement: Supplementary file 1 [file plants-11-01143-s001.zip › plants-1677615-supplementary.pdf]

plants

MDPI

Supplementary Materials

Chemical and molecular characterization of wound-induced suberization in poplar (*Populus alba* × *P. tremula*) stem bark

Meghan K. Rains[1,2], Christine Caron[1], Sharon Regan[2] and Isabel Molina[1,*]

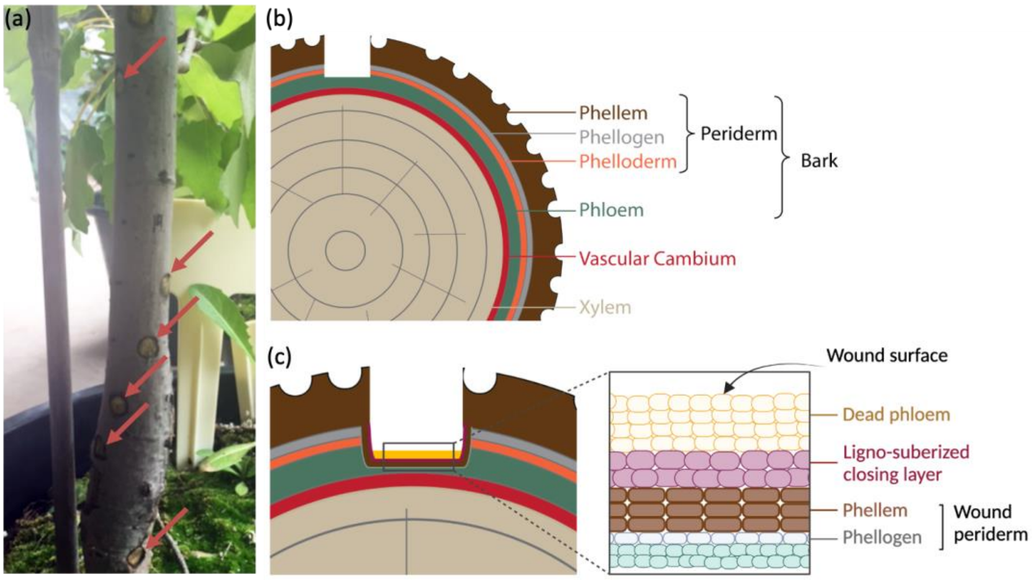

Figure S1. Wounding approach and wound periderm formation. (a) Representative wounded tree showing removed bark periderm. Sixteen 7 mm-diameter punches from hybrid poplar bark were removed from each tree. Red arrows indicate where samples that are seen in this picture were taken. (b) Schematic representing the wound infringed on the poplar bark (to a 1 mm depth from the edge). (b) Diagram representing the ligno-suberized closing layer formed by suberization of existing phloem cells and a new wound periderm developed internally to the closing layer. Diagrams shown in (b) and (c) were created with BioRender.com.

Table S1. Wax components extracted by chloroform immersion at days 0-14 post wounding. The average from three biological replicates and SD are reported; concentration units are μg/cm². MAG: monoacylglycerols.

Day post wounding	Fatty Acids	SD	Primary Alcohols	SD	Sterols	SD	MAG	SD
0	14.8	2.6	1.9	0.9	12.9	11.5	0.0	0.0
1	18.3	3.2	2.8	0.4	36.0	9.6	2.7	2.4
2	30.8	4.2	5.2	1.0	32.9	5.6	7.6	5.0
3	18.8	2.8	3.1	1.2	15.4	11.6	2.4	0.8
4	18.8	4.3	3.4	0.7	17.2	3.2	2.0	0.7
5	9.0	0.8	1.7	0.6	8.9	6.3	0.9	0.2
6	14.9	2.7	3.6	0.5	15.1	3.9	1.8	0.5
7	10.7	1.9	5.4	2.2	12.6	3.2	2.6	0.5
8	37.4	4.3	4.9	3.5	15.4	0.7	4.7	2.2
9	15.2	4.2	5.1	1.6	12.5	9.6	1.7	0.7
10	14.5	2.0	7.3	2.2	17.5	5.9	2.2	0.4
11	15.8	2.7	14.6	5.4	21.4	0.7	3.6	0.7
12	25.2	4.3	9.7	4.6	17.6	2.0	3.4	0.9
13	18.7	2.7	11.4	6.5	15.8	0.9	2.5	0.7
14	14.7	4.9	4.4	1.4	10.5	3.5	1.3	0.3

Table S2. Suberin monomer composition (mole %) at days 0-14 post wounding. The average from three biological replicates and SE are reported; concentration units are μmol/cm². HCAs: hydroxycinnamic acids.

		Day post wounding															
		**Day 0**		**Day 1**		**Day 2**		**Day 3**		**Day 4**		**Day 5**		**Day 6**		**Day 7**	
**Suberin monomer**		**Mol %**	**SE**	**Mol %**	**SE**	**Mol %**	**SE**	**Mol %**	**SE**	**Mol %**	**SE**	**Mol %**	**SE**	**Mol %**	**SE**	**Mol %**	**SE**
**HCAs**	**Coumaric Acid *cis***	10.9	2.3	8.8	2.7	6.0	1.9	7.1	2.0	10.3	1.5	7.6	2.0	5.8	1.5	5.8	1.0
	**Coumaric Acid *trans***	30.9	10.5	37.1	14.7	32.5	11.4	32.8	8.8	39.8	7.0	18.0	4.1	22.8	6.9	23.3	7.9
	**Ferulic Acid**	3.0	1.0	2.3	0.6	2.7	1.3	2.5	0.40	3.2	1.2	4.5	0.6	3.2	0.6	3.4	0.4
	**Total**	44.9	10.8	48.1	14.9	41.2	11.6	42.4	9.0	53.3	7.3	30.0	4.6	31.9	7.1	32.5	8.0
**Fatty Acids**	**16:0**	24.2	3.3	16.7	4.9	10.9	1.7	16.8	7.3	13.1	1.5	12.3	3.0	13.3	2.6	12.6	2.5
	**18:0**	14.6	4.6	7.5	2.8	4.9	1.3	8.1	5.6	4.1	1.1	4.3	1.0	4.5	1.2	7.9	3.0
	**20:0**	0.0	0.0	0.0	0.0	0.0	0.0	0.0	0.0	0.0	0.0	0.0	0.0	0.7	0.1	1.0	0.3
	**22:0**	0.0	0.0	2.0	0.8	3.6	1.2	2.8	0.1	7.1	4.4	5.8	0.4	20.4	15.6	6.0	0.9
	**24:0**	0.0	0.0	0.5	0.5	1.4	0.6	1.2	0.3	0.7	0.2	1.4	0.3	0.7	0.2	1.1	0.1
	**Total**	38.8	5.6	26.8	5.7	20.8	2.5	28.9	9.2	25.0	4.8	23.7	3.2	39.5	15.8	28.5	4.0
**Dicarboxylic Acids**	**16:0**	8.5	3.2	9.3	2.9	12.7	3.7	10.9	0.5	8.8	2.3	17.9	1.4	13.1	3.9	18.2	1.6
	**18:1**	0.7	0.7	2.7	1.3	4.8	2.5	3.2	0.8	2.3	0.7	5.5	0.9	3.5	1.5	5.5	0.5
	**18:0**	0.0	0.0	0.0	0.0	0.0	0.0	0.0	0.0	0.0	0.0	0.8	0.1	0.3	0.3	0.9	0.1
	**20:0**	0.0	0.0	0.0	0.0	0.0	0.0	0.0	0.0	0.0	0.0	0.0	0.0	0.0	0.0	0.0	0.0
	**22:0**	0.0	0.0	0.0	0.0	0.0	0.0	0.0	0.0	0.0	0.0	0.0	0.0	0.0	0.0	0.0	0.0
	**Total**	9.2	3.3	12.0	3.2	17.5	4.5	14.2	1.0	11.1	2.4	24.1	1.6	16.9	4.2	24.7	1.7
**Hydroxy Fatty Acids**	**16-OH 16:0**	6.4	1.7	9.7	2.8	13.1	4.3	10.0	1.0	8.0	1.2	14.8	5.3	8.5	3.6	9.3	0.8
	**18-OH 18:1**	0.7	0.7	2.9	1.3	6.3	3.7	3.6	1.1	1.6	0.5	5.3	2.3	2.1	1.1	3.3	0.2
	**18-OH 18:0**	0.0	0.0	0.0	0.0	0.0	0.0	0.0	0.0	0.0	0.0	0.0	0.0	0.0	0.0	0.0	0.0
	**20-OH 20:0**	0.0	0.0	0.0	0.0	0.0	0.0	0.0	0.0	0.0	0.0	0.0	0.0	0.0	0.0	0.0	0.0
	**22-OH 22:0**	0.0	0.0	0.5	0.5	1.2	0.5	1.0	0.1	1.1	0.3	1.9	0.2	1.1	0.4	1.7	0.3
	**24-OH 24:0**	0.0	0.0	0.0	0.0	0.0	0.0	0.0	0.0	0.0	0.0	0.2	0.2	0.0	0.0	0.0	0.0

	**Total**	7.1	1.8	13.1	3.1	20.5	5.7	14.6	1.5	10.7	1.4	22.1	5.7	11.7	3.7	14.3	0.9
**Fatty Alcohols**	**1-OH 20:0**	0.0	0.0	0.0	0.0	0.0	0.0	0.0	0.0	0.0	0.0	0.0	0.0	0.0	0.0	0.0	0.0
	**1-OH 22:0**	0.0	0.0	0.0	0.0	0.0	0.0	0.0	0.0	0.0	0.0	0.0	0.0	0.0	0.0	0.0	0.0
	**Total**	0.0	0.0	0.0	0.0	0.0	0.0	0.0	0.0	0.0	0.0	0.0	0.0	0.0	0.0	0.0	0.0

Table S2 (Continued). Suberin monomer composition (mole %) at days 0-28 post wounding. The average from three biological replicates and SE are reported; concentration units are μmol/cm^2. HCAs: hydroxycinnamic acids.

		**Day post wounding**													
		**Day 8**		**Day 9**		**Day 10**		**Day 11**		**Day 12**		**Day 13**		**Day 14**	
**Suberin monomer**		**Mol %**	**SE**	**Mol %**	**SE**	**Mol %**	**SE**	**Mol %**	**SE**	**Mol %**	**SE**	**Mol %**	**SE**	**Mol %**	**SE**
**HCAs**	**Coumaric Acid *cis***	7.0	0.6	5.1	0.7	2.8	0.7	2.9	0.4	2.3	0.7	1.2	0.2	1.5	0.5
	**Coumaric Acid *trans***	35.2	1.9	20.5	3.8	13.7	4.1	12.8	2.2	13.7	5.9	6.4	1.6	6.7	2.6
	**Ferulic Acid**	3.5	0.5	3.7	0.4	2.7	0.6	3.4	0.3	3.4	0.5	2.8	0.2	3.0	0.1
	**Total**	45.7	2.1	29.3	3.8	19.2	4.2	19.1	2.3	19.4	5.9	10.4	1.6	11.2	2.6
**Fatty Acids**	**16:0**	12.9	1.2	8.5	1.1	7.8	0.1	7.6	1.8	5.6	0.7	6.6	1.0	4.6	0.8
	**18:0**	3.8	0.5	1.7	0.2	2.5	0.8	1.9	0.5	1.8	0.3	1.4	0.3	1.0	0.0
	**20:0**	0.7	0.1	0.6	0.0	0.9	0.0	0.6	0.1	0.6	0.1	0.5	0.0	0.6	0.1
	**22:0**	4.1	0.0	5.0	0.5	12.5	7.4	5.9	0.4	5.6	0.5	6.3	1.0	7.1	0.7
	**24:0**	0.6	0.0	0.7	0.1	1.1	0.1	0.9	0.1	0.7	0.1	0.9	0.1	1.0	0.1
	**Total**	22.2	1.3	16.6	1.3	24.8	7.4	17.0	1.9	14.3	0.9	15.7	1.5	14.3	1.0
**Dicarboxylic Acids**	**16:0**	17.3	1.7	23.3	0.8	23.5	1.4	25.7	0.9	25.1	1.1	27.9	0.4	27.7	0.8
	**18:1**	4.5	0.5	8.8	0.3	11.0	0.4	10.1	1.0	12.0	1.6	12.3	0.4	12.1	0.7
	**18:0**	0.7	0.1	1.0	0.1	1.1	0.0	1.2	0.1	1.4	0.1	1.2	0.1	1.5	0.2
	**20:0**	0.0	0.0	0.1	0.0	0.2	0.1	0.2	0.0	0.2	0.1	0.2	0.0	0.2	0.1

	**22:0**	0.0	0.0	0.3	0.0	0.5	0.1	0.3	0.0	0.3	0.1	0.4	0.1	0.4	0.0
	**Total**	22.6	1.7	33.5	0.9	36.3	1.5	37.4	1.3	39.1	1.9	42.0	0.6	41.9	1.0
**Hydroxy Fatty Acids**	**16-OH 16:0**	6.4	0.7	12.8	1.3	11.8	1.8	15.9	1.6	14.9	3.1	18.3	0.7	18.8	1.0
	**18-OH 18:1**	2.0	0.3	5.8	0.7	6.2	1.1	7.6	0.9	9.3	2.3	9.2	0.4	9.0	0.3
	**18-OH 18:0**	0.0	0.0	0.0	0.0	0.0	0.0	0.2	0.0	0.2	0.0	0.1	0.0	0.2	0.1
	**20-OH 20:0**	0.0	0.0	0.3	0.0	0.2	0.0	0.2	0.0	0.3	0.1	0.3	0.0	0.3	0.0
	**22-OH 22:0**	1.2	0.1	1.8	0.2	1.8	0.4	2.2	0.2	2.4	0.2	2.8	0.4	3.1	0.3
	**24-OH 24:0**	0.0	0.0	0.0	0.0	0.0	0.0	0.0	0.0	0.0	0.0	0.2	0.0	0.2	0.0
	**Total**	9.5	0.8	20.7	1.5	20.0	2.2	26.1	1.9	27.0	3.8	30.9	0.9	31.7	1.1
**Fatty Alcohols**	**1-OH 20:0**	0.0	0.0	0.0	0.0	0.0	0.0	0.0	0.0	0.0	0.0	0.4	0.0	0.5	0.1
	**1-OH 22:0**	0.0	0.0	0.0	0.0	0.0	0.0	0.4	0.0	0.2	0.1	0.5	0.1	0.5	0.1
	**Total**	0.0	0.0	0.0	0.0	0.0	0.0	0.4	0.0	0.2	0.1	0.9	0.1	1.0	0.2

Optimization of a Stable Isotope Dilution Assay for Glycerol Quantification in Poplar Periderm Suberin.

The chemical composition of suberin ester-bound aliphatics shown in Figure 2 c was determined using base-catalyzed depolymerization. However, this and other methods of chemical depolymerization of suberized tissues use organic solvent-aqueous phase partitioning to extract the organic solvent-soluble monomers; because glycerol is hydrophilic, it is typically discarded with the aqueous phase and not quantified in such protocols. Glycerol is a major suberin monomer, and is widely believed to provide the backbone necessary to facilitate the assembly of the aliphatic domain of suberin [1–3]. Previous studies of glycerol content in both cutin and suberin have used chemical and enzymatic methods to quantify the monomer [3–10]. Enzymatic methods are expensive, and as a result, high throughput analysis is not practical. Chemical methods are limited by glycerol losses in the aqueous phase during solvent partition, or the evaporation step when low boiling and chemically dissimilar standards such as glyceraldehyde or 1,2-propanediol are used. The use of 1,12-dodecanediol has showed some success as an internal standard for glycerol quantification [7], as it is less volatile; however the resulting derivatized structure is dissimilar to glycerol, which can impact the accuracy of the amount of glycerol quantified. However, a labelled internal standard, which has the same physiochemical properties as the monomer being quantified, accounts for any losses that may occur throughout the depolymerization procedure, leading to a more accurate determination of the amount of glycerol present [11]. The stable isotope dilution method allows for the simultaneous determination of glycerol and acyl-monomers using the same sample, allowing for greater confidence in the stoichiometric relationships derived, which can be used to make structural inferences.

Following the method described by Yang and co-workers, glycerol in the suberin polymer was quantified using the internal standard [$^{13}C_3$]-glycerol, which gives a unique isotopomer signal three mass units higher when analyzed by GC-MS. The use of diagnostic ions 115/116 and 118/119 for the acetylated glycerol (triacetin) and acetylated [$^{13}C_3$]-glycerol ([$^{13}C_3$]glyceryl)triacetin; Figure S2 a) respectively, allowed us to generate calibration curves comparing the ratio of ion current between the two (i.e. 115/118 and 116/119), showing that the measured values were a linear function of the molar ratio of glycerol and $^{13}C_3$-glycerol (Figure S2 a). Each ion pair gave R^2 values >0.99, and therefore no significant differences in using either pair was determined. In an effort to be consistent with the published method for cutin glycerol, the 116/119 ion pair was selected for glycerol quantification [11]

Although similar, cutin and suberin are not identical and therefore we adapted the methodology to depolymerize suberin and quantitatively determine glycerol and aliphatic monomer content in the polyester. For *Arabidopsis* stem cutin, which is known to be rich in dicarboxylic acids, a concentration of 0.2% (w/v) sodium methoxide (NaOMe) was sufficient to depolymerize cutin [11]. Due to the complex structure of suberin, we first optimized the concentration of NaOMe on a 10 mg sample of tree bark to ensure complete depolymerization. Concentrations of 0.2%, 0.5% 1% and 6% were chosen as they cover a wide range, including 6%, which is the standard amount used in sodium methoxide-catalyzed depolymerizations [12]. As a consequence of the lack of solvent partitioning steps, using a minimum amount of catalyst was important for downstream isolation of glycerol and suberin monomers, and also helped to minimize the overall volume to be evaporated. In our hands, 0.2% (w/v) NaOMe failed to liberate significant amounts of suberin monomers (data not shown). As illustrated in Figure S2 b, each of the other concentrations examined liberated approximately equal proportions of the major monomer classes; FAs, α,ω-DCAs and ω-OHFAs. Compositionally, the standard 6% (w/v) catalyst concentration yielded molar percentages of 2.7±0.05 %, 36.0±2.4% and 61.3±4.9% for FAs, α,ω-DCAs and ω-OHFAs respectively, with no significant changes in the molar composition determined between the 1% and 6% samples. The lowest catalyst concentrations (0.5%), appeared to preferentially liberate α,ω-DCAs at the expense of ω-OHFAs, and was therefore deemed unsuitable for analysis of poplar bark aliphatic suberin due to the abundance of ω-OHFA monomers [13].

The internal standard ω-pentadecanolactone was added as a control to monitor the completeness of both the transmethylation of primary esters and completeness of derivatization [11,12,14].

At each concentration assayed, this lactone was recovered as the acetylated 15-hydroxy pentadecanoate derivative, indicating that even the 0.5% concentration effectively cleaved primary ester bonds. Although approximately 10% of DCAs are lost at high concentrations compared to 0.5%, we selected 1% (w/v) NaOMe for the optimized suberin glycerol analysis because it achieves the same results as the conventional method, which uses 6% NaOMe (Figure S2 b).

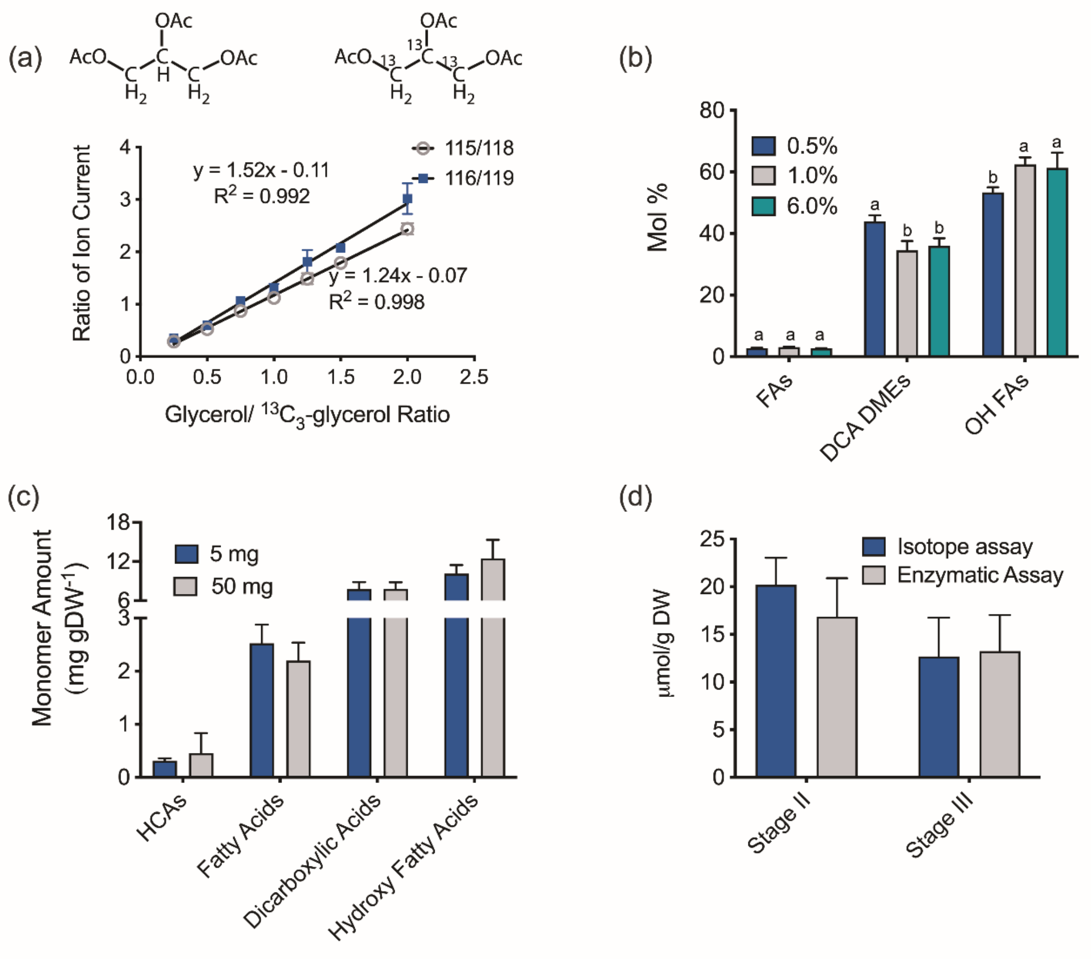

Figure S2. Optimization of a stable isotope dilution assay for glycerol quantification in poplar periderm suberin. (a) Structures of $^{12}C_3$-triacetin and $^{13}C_3$-triacetin and calibration plot of ratio of ion current to ratio of the amount of triacetin and [$^{13}C_3$]-triacetin at the indicated masses. Values are expressed as the mean of a minimum of 4 replicates ± SD. **(b)** Optimization of catalyst concentration. Percent of total molar values (% mol) are expressed as the mean of a minimum of 4 replicates ± SD. Statistics determined for each monomer class using one-way ANOVA and Tukey post-hoc test, $p<0.05$. **(c)** Optimization of tissue amounts. Amount of each monomer class liberated from stage III bark tissues determined using 6% NaOMe catalyst. Values are expressed as the mean of a minimum of 4 replicates ± SD. No significant differences were found between each amount, t-test, $p < 0.05$. **(d)** Comparison of glycerol yields using enzymatic determination and $^{13}C3$-glycerol dilution assay. Glycerol was quantified using a commercial kit that uses a coupled enzyme assay involving glycerol kinase and glycerol phosphate oxidase and compared to results of isotope dilution assay. Means of a minimum of 4 replicates ± SE are shown.

Following optimization of catalyst concentration, we then optimized the amount of tissue depolymerized. Previous depolymerizations of poplar bark tissues have used 50 mg of cell wall enriched dry material [13], whereas Yang and co-workers (2016) were able to achieve reproducible results with 30 mg of dried *Arabidopsis* plant materials. In our hands, these large tissue amounts

lead to difficulties with monomer recovery, due to crystalized material on the walls of the container. Tissue amounts varying from 5-50 mg (5, 10, 30 and 50 mg) were depolymerized with a 1% catalyst concentration (results not shown), however large variations in monomer recoveries were noted, with only 15% of the expected amounts recovered when 30 and 50 mg were used. Consequently, a standard depolymerization approach (6% NaOMe) was used to determine reaction efficiency when lower tissue amounts were used. A similar approach was taken by the original authors for all optimization steps [11]. As illustrated in Figure S2 c, no significant differences in monomer recoveries were found when comparing depolymerizations of stage III bark tissues using 5 mg and 50 mg under standard depolymerization conditions. The total amount of monomers liberated from 5 mg was found to be 20.8 ± 2.8 mg gDW^{-1} whereas 50 mg of tissue liberated 23.1 ± 3.6 mg gDW^{-1}. Similar results were found for stage II tissues, with the 5 mg sample liberating 29.5 ± 1.6 mg gDW^{-1} and the 50 mg sample released 24.2 ± 7.8 mg gDW^{-1}. Therefore, to minimize monomer losses which occurred at higher tissue amounts, 5 mg was selected for the optimized protocol. As no changes were made to the reaction time, this step was not optimized for suberin depolymerization, and a standard length of 2 hours was used.

To further validate the stable isotope dilution methodology in suberin, glycerol was quantified using a commercially available kit (MAK117, Sigma-Aldrich) that uses an enzymatic assay involving glycerol kinase and glycerol phosphate oxidase to generate a colorimetric product monitored at 570 nm. Tissues were delipidated, depolymerized, recovered and dried down according to the optimized method to simultaneously determine glycerol and aliphatic monomers described above. Once no trace of solvent could be detected, residues were resuspended in water, filtered and concentrated before a final suspension in sterile water. Our results, outlined in Figure S2 d indicate that the $^{13}C_3$-glycerol dilution assay quantified glycerol in samples as well as conventional enzymatic methods.

Modified Microscale Green Solvent Thioacidolysis Assay

Thioacidolysis is a routinely applied method to determine H/G/S monolignol content in lignified tissues and has been used to characterize the aromatic domain of suberin. This method selectively cleaves 8-O-4′ ethers and is considered a more accurate analytical technique than other methods that analyze total aromatic content, however, it uses dioxane as a solvent. Dioxane is a toxic solvent, which is capable of forming explosive peroxides over time, is environmentally unfriendly and is suspected of causing cancer (Derosa et al. 1996; Capello et al. 2007). The main goal of thioacidolysis is to provide relative monomer proportions rather than absolute monomer quantification, therefore underestimation of the absolute yield of 8-O-4′-bound monolignols is not a concern. Robinson and Mansfield (2009) were the first to adapt the original thioacidolysis protocols [15,16] by reducing the reaction volume to facilitate high throughput analysis without specialized equipment or unnecessary washing steps. This was later modified by Foster and coworkers (2010) who further reduced the reaction volume and sample size to permit a microscale analysis of cell wall isolated dry material. We have utilized a combination of these two methods to develop a green-solvent approach to thioacidolysis. By substituting dioxane with CPME in the reaction, we are able to carry out high throughput analysis of the suberin aromatic domain using very little material and significantly reducing the toxicity of the overall reaction. Furthermore, by eliminating the use of dichloromethane in the chemical work up, we have eliminated a second possible carcinogen and replaced it with a more benign alternative.

To validate the modified method, we applied the original thioacidolysis protocol and our modified approach to poplar wood and bark as well as to potato periderm samples (Figure S3). Although this reaction is typically run for 4 hours under standard conditions, 30 minutes was sufficient to produce extensive monolignol amounts at a ratio of approximately 70:30 S:G in poplar wood samples (Figure S3 a). This ratio was maintained across all time points and both solvents, indicating the suitability of CPME to replace dioxane in thioacidolysis reactions to analyze monolignol composition. The most consistent results were obtained at 4 hours; both solvents released approximately equal amounts of monomers at the same ratio with good reproducibility, and therefore this reaction time was selected for further study. Although the dioxane protocol liberates monolignol monomers more efficiently than the CPME protocol, thioacidolysis using either

solvent sufficiently released enough monolignol to accurately quantify their relative abundance (Figure S3 b). As with all thioacidolysis methods, the yield is always an underestimate of the total lignin content, and our only goal here was to evaluate monomer proportions [17]. The relative proportions of S:G with both solvent systems were 70:30 for wood (consistent with results reported by [17], and 50:50 S:G for bark stages II (4.0-5.5 mm stem diameter) and stage III (6.0-8.0 mm diameter) as defined in [13] (Figure S3 c). The greatest variability among solvents was noted in the potato periderm tissues where dioxane found a 30:70 ratio while CPME established a 20:80 S:G ratio (Figure S3 c). The results obtained using dioxane agree well with [18] who found a relative abundance of 35:65 S:G in natural potato periderms. Small variations in the S:G ratios of potato may be the result of cultivar selected.

Much like previous attempts, although many procedural iterations were attempted (i.e. reaction volume, time, tissue amount, chemical work up), we were only able to implement small changes which made little impact on the efficiency of the traditional methodology. We were unable to further reduce reaction volume while maintaining accuracy, although, as outlined by [19]sample size could be reduced to 2 mg from 10 mg and the reaction clean up procedure no longer requires halogenated solvents. Additionally, we have utilized a more efficient derivatization procedure to minimize the time required for each sample. It has been reported that as little as 200 μL of reaction mixture can be used with 2 mg of tissue [19], however in our hands 1 mL was the minimum reaction volume that would generate reproducible data.

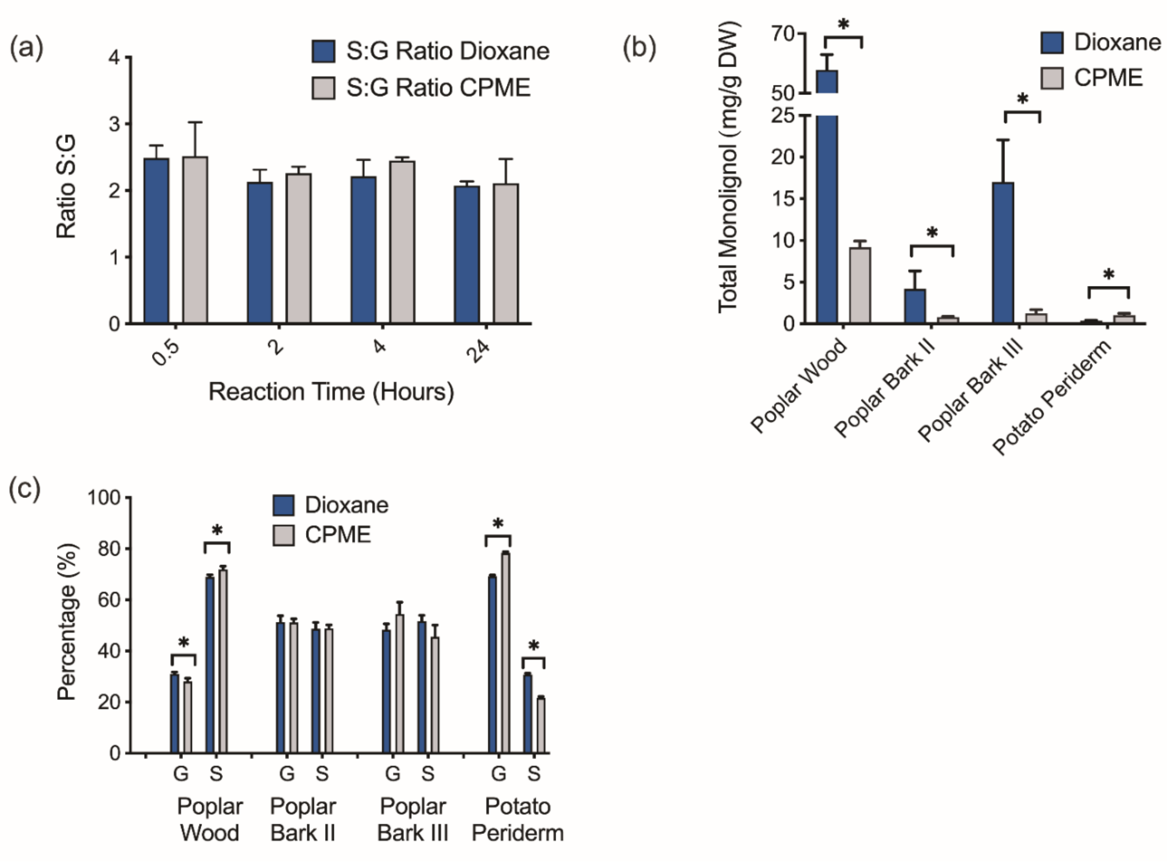

Figure S3. Modified thioacidolysis method validation. (a) S:G ratio in wood samples as a function of reaction time with each solvent. Values expressed as the mean of 4 replicates ± SD. Dioxane 24 hour time point reported as average of 2 replicates. No significant differences were found between each time point, t-test, $p < 0.05$. **(b)** Monolignol amounts quantified using tetracosane as an internal standard with each solvent system. **(c)** Relative percentage of each monolignol in various tissues and each solvent system. G refers to guaiacyl units, and S refers to syringyl units. Values are expressed as the mean of 4 replicates ± SD. (*) denotes significance, t-test $p < 0.05$.

References

1. Bernards, M.A. Demystifying suberin. *Can. J. Bot.* **2002**, *80*, 227–240, doi:10.1139/b02-017.

2. Graça, J.; Pereira, H. Glyceryl-acyl and aryl-acyl dimers in *Pseudotsuga Menziesii* bark suberin. *Holzforschung* **1999**, *53*, 397-402.

3. Moire, L.; Schmutz, A.; Buchala, A.; Yan, B.; Stark, R.E.; Ryser, U. Glycerol is a suberin monomer. new experimental evidence for an old hypothesis. *Plant Physiol.* **1999**, *119*, 1137–1146.

4. Graça, J.; Pereira, H. Cork Suberin: a glyceryl based polyester. *Holzforschung* **1997**, *51*, 225–234.

5. Graça, J.; Pereira, H. Feruloyl Esters of Omega-Hydroxyacids in Cork Suberin. *J. Wood Chem. Technol.* **1998**, *18*, 207–217.

6. Graça, J.; Pereira, H. Diglycerol alkenedioates in suberin: building units of a poly(acylglycerol) polyester. *Biomacromolecules* **2000**, *1*, 519–522.

7. Graça, J.; Pereira, H. Methanolysis of bark suberins: analysis of glycerol and acid monomers. *Phytochem. Anal.* **2000**, *11*, 45–51.

8. Graça, J.; Pereira, H. Suberin structure in potato periderm: glycerol, long-chain monomers, and glyceryl and feruloyl dimers. *J. Agric. Food Chem.* **2000**, *48*, 5476–5483.

9. Schmutz, A.; Buchala, A.J.; Ryser, U. Changing the dimensions of suberin lamellae of green cotton fibers with a specific inhibitor of the endoplasmic reticulum-associated fatty acid elongases. *Plant Physiol.* **1996**, *110*, 403–411.

10. Schmutz, A.; Jenny, T.; Amrhein, N.; Ryser, U. Caffeic acid and glycerol are constituents of the suberin layers in green cotton fibres. *Planta* **1993**, *189*, 453–460.

11. Yang, W.; Pollard, M.; Li-Beisson, Y.; Ohlrogge, J. Quantitative analysis of glycerol in dicarboxylic acid-rich cutins provides insights into Arabidopsis cutin structure. *Phytochemistry* **2016**, 1–11.

12. Molina, I.; Bonaventure, G.; Ohlrogge, J.; Pollard, M. The Lipid polyester composition of *Arabidopsis thaliana* and *Brassica napus* seeds. *Phytochemistry* **2006**, *67*, 2597–2610.

13. Rains, M.K.; de Silva, N.D.G.; Molina, I. reconstructing the suberin pathway in poplar by chemical and transcriptomic analysis of bark tissues. *Tree Physiol.* **2018**, *38*, 340–361.

14. Bonaventure, G.; Beisson, F.; Ohlrogge, J.; Pollard, M. Analysis of the Aliphatic Monomer Composition of Polyesters Associated With Arabidopsis Epidermis: Occurrence of Octadeca-Cis-6, Cis-9-Diene-1,18-Dioate as the Major Component. *The Plant Journal* **2004**, *40*, 920–930.

15. Lapierre, C.; Monties, B.; Rolando, C.; de Chirale, L. Thioacidolysis of lignin: comparison with acidolysis. *J. Wood Chem. Technol.* **1985**, *5*, 277–292.

16. Rolando, C.; Monties, B.; Lapierre, C. Thioacidolysis. In *Methods in Lignin Chemistry* Lin, S.Y., Dence, C.W. (Eds). Springer Series in Wood Science. Springer, Berlin, Heidelberg, pp. 334–349.

17. Robinson, A.R.; Mansfield, S.D. Rapid analysis of poplar lignin monomer composition by a streamlined thioacidolysis procedure and near-infrared reflectance-based prediction modeling. *Plant J.* **2009**, *58*, 706–714.

18. Lapierre, C.; Pollet, B.; Négrel, J. The phenolic domain of potato suberin: structural comparison with lignins. *Phytochemistry* **1996**, *42*, 949–953.

19. Foster, C.E.; Martin, T.M.; Pauly, M. Comprehensive compositional analysis of plant cell walls (Lignocellulosic Biomass) Part I: Lignin. *JOVE* **2010**, 1–4, web doi:10.3791/1745.
